# Single-cell RNA sequencing identifies M2-like macrophage polarization associated with mesenchymal stem cell treatment in a murine sepsis model

**DOI:** 10.17305/bb.2026.13517

**Published:** 2026-02-20

**Authors:** Takehiko Oami, Takahisa Hishiya, Seiji Miyauchi, Chiaki Iwamura, Kiyoshi Hirahara, Taka-aki Nakada

**Affiliations:** 1 Department of Emergency and Critical Care Medicine, Chiba University Graduate School of Medicine, Chiba, Japan; 2 Department of Immunology, Graduate School of Medicine, Chiba University, Chiba, Japan; 3 Department of Orthopaedic Surgery, Graduate School of Medicine, Chiba University, Chiba, Japan

**Keywords:** Cecal ligation and puncture, gene expression, macrophage polarization, pseudotime trajectory analysis, critical care

## Abstract

Mesenchymal stem cells (MSCs) have demonstrated therapeutic potential in preclinical models of sepsis, primarily through their immunomodulatory functions. However, the specific mechanistic roles of MSCs in the pathophysiology of sepsis, particularly their interactions with immune cells, are not yet fully understood. In this study, we utilized a murine sepsis model induced by cecal ligation and puncture to assess the effects of adipose-derived MSCs on survival rates, systemic cytokine profiles, and immune cell dynamics. MSCs were administered via tail vein injection immediately after surgery. To investigate cell-specific transcriptional changes and associated pathway enrichment following MSC administration, we performed single-cell RNA sequencing (scRNA-seq) on CD45-positive immune cells isolated six hours postoperatively. Our results indicated that MSC administration significantly improved the survival of septic mice compared to controls. The scRNA-seq analysis revealed dynamic changes in immune cell populations, including increased proportions of both M1 and M2 macrophages. Transcriptomic analysis demonstrated that MSC treatment downregulated inflammatory genes such as *Cd14*, *Cxcl2*, *Hmgb2*, and *Pde4b* in M0 and M1 macrophages while upregulating regulatory and metabolic genes, including *Hmox1*, *Maf*, and *Jun*. In M2 macrophages, MSCs enhanced the expression of immunomodulatory genes such as *Hmox1* and *Ccr1*, indicating a potential promotion of inflammation resolution. Pseudotime trajectory analysis suggested a transition towards M2-like transcriptional states. Overall, MSC treatment resulted in improved survival in sepsis, characterized by immune modulation, reduced inflammatory signatures, and increased M2-like macrophage profiles. These findings offer mechanistic insights into the therapeutic potential of MSCs and underscore the need for further research to optimize their clinical applications in sepsis.

## Introduction

Sepsis is a life-threatening condition characterized by organ dysfunction due to a dysregulated host response to infection, resulting in approximately 11 million deaths globally each year [1, 2]. Despite the substantial global burden of sepsis, no targeted therapies are currently available to directly address its underlying pathophysiology. While numerous anti-inflammatory agents have been assessed for their potential to mitigate excessive immune activation, clinical trials have consistently failed to replicate the therapeutic efficacy observed in animal models [3]. This discrepancy highlights the urgent need for innovative strategies to develop treatments that can effectively modulate sepsis pathogenesis.

Mesenchymal stem cells (MSCs) are multipotent progenitor cells capable of differentiating into stromal lineages, including bone and cartilage [4, 5]. Extensive research has demonstrated the therapeutic potential of MSCs across a variety of diseases, such as ischemic heart disease, inflammatory bowel disease, and spinal cord injury, due to their regenerative and immunomodulatory properties [6]. In sepsis, preclinical studies involving animal models have reported that MSC administration can exert anti-inflammatory effects and enhance survival [7]. Building on these promising findings, phase I clinical trials are currently underway in several countries [8].

The therapeutic effects of MSCs in conditions beyond sepsis are attributed to mechanisms including the production of anti-inflammatory cytokines and the secretion of extracellular vesicles [5]. A previous study demonstrated that MSCs enhance the anti-inflammatory activity of macrophages via the prostaglandin E2 pathway in a mouse model of sepsis [9]. However, the precise mechanisms through which MSCs exert their effects in sepsis remain to be fully elucidated. Although recent advances in single-cell RNA sequencing (scRNA-seq) technologies have provided deeper insights into the functional heterogeneity of immune cells [10–13], these methodologies have rarely been applied in MSC sepsis models, and to our knowledge, have not yet been utilized in a cecal ligation and puncture (CLP) model with systemic CD45^+^ immune profiling.

We hypothesized that MSCs ameliorate sepsis pathophysiology through complex interactions with immune cells. Thus, this study aimed to elucidate the mechanistic role of MSCs in a murine model of sepsis using scRNA-seq.

## Materials and methods

### Mice

Experiments were conducted using 8- to 12-week-old male C57 BL/6 mice obtained from CLEA Japan, Inc. (Tokyo, Japan). All mice were acclimatized for a minimum of one week prior to experimentation and were housed under specific pathogen-free conditions in a controlled environment with regulated temperature, humidity, and a 12-hour light/dark cycle.

### CLP operation

Abdominal sepsis was induced using the CLP method as previously described [14]. Under isoflurane anesthesia, a midline incision was made to expose the cecum. The fecal content was gently moved toward the cecal tip, and the cecum was ligated at the midpoint between the tip and the ileocecal valve using a 4-0 silk suture. The ligated cecum was punctured twice using a 25-gauge needle, ensuring full penetration and allowing a small amount of stool to be extruded. The rectus abdominis was sutured, and the skin was sealed with glue. Following surgery, 1 mL of normal saline was administered subcutaneously for fluid resuscitation. For all experiments, ceftriaxone (50 mg/kg; NIPRO, Osaka, Japan) and metronidazole (35 mg/kg; Pfizer Japan, Tokyo, Japan) were administered subcutaneously every 12 hours for 48 hours to simulate clinical treatment conditions. Analgesics were not administered during the experiments to avoid confounding effects on immune responses in the CLP model. Animals were closely monitored, and humane endpoints were applied in accordance with institutional guidelines. The experimental conditions were optimized to create a clinically relevant sepsis model targeting a mortality rate of approximately 50% at 168 hours post-CLP surgery.

### MSCs

Adipose-derived MSCs were isolated from human donors through enzymatic digestion, followed by centrifugation. MSCs, provided by ROHTO Pharmaceutical Co., Ltd., were cultured in the company’s proprietary medium (ROHTO Pharmaceutical Co., Ltd., Kyoto, Japan). Human adipose tissue utilized for MSC manufacturing was obtained by ROHTO Pharmaceutical Co. under institutional review board-approved protocols with written informed consent from donors, as described in previously published research using the same MSC product [15–17]. The MSCs used in this study met established criteria for MSCs, including the expression of positive markers and the absence of negative markers, as previously defined [18]. The same lot of adipose-derived MSCs from ROHTO Pharmaceutical Co., Ltd. was utilized throughout the study. Cryopreserved MSCs were thawed rapidly in a 37 ^∘^C water bath, washed with culture medium to remove cryoprotectants, and resuspended for subsequent culture or analysis under sterile conditions. Human adipose-derived MSCs (AD-MSCs) were obtained from ROHTO, used at passage 4, thawed immediately prior to injection, and confirmed to have >80% viability. The treatment group was randomly assigned at the cage level to receive 1×10^6^ MSCs via the tail vein immediately following surgery, while the control group received vehicle buffer using the same method. Blinding of outcome assessment was not performed due to practical constraints; however, objective endpoints and standardized procedures were employed to minimize potential bias.

### Survival study

Survival following CLP surgery was monitored every 12 hours for a period of 168 hours. Mice that reached ethical endpoints, defined as severe weakness (e.g., inability to consume food or water), signs of distress, or sustained weight loss exceeding 20% over several days, were euthanized.

### Cytokine analysis

Plasma samples obtained 6 hours after the CLP procedure were analyzed to measure levels of interleukin (IL)-1α, IL-1β, IL-6, IL-10, IL-12 p70, IL-13, tumor necrosis factor (TNF)-α, interferon gamma (IFN-γ), granulocyte-macrophage colony-stimulating factor (GM-CSF), monocyte chemotactic protein-1 (MCP-1), macrophage inflammatory protein-1 alpha (MIP-1α), and macrophage inflammatory protein-1 beta (MIP-1β). Cytokine concentrations were determined using the Bio-Plex 200 System (Bio-Rad, Hercules, CA, USA) and the Bio-Plex Pro Mouse Cytokine 23-Plex Panel (Bio-Rad).

### scRNA-seq

Blood samples were collected from the mice 6 hours postoperatively, and immune cells (CD45-positive) were isolated using magnetic beads. The samples were subsequently encapsulated into droplets, the cells were lysed, and cDNA was synthesized using the Chromium Single Cell 3.1 Reagent Kits v3 to create a library with an index sequence for each cell, following the manufacturer’s instructions (10x Genomics, Pleasanton, CA, USA). The resulting scRNA-seq libraries were sequenced using a NovaSeq 6000 (Illumina, San Diego, CA, USA) with 128-cycle paired-end reads [19]. Individual 10x Genomics libraries were generated for each mouse (*n* ═ 3 per group) and sequenced separately.

### Bioinformatics for scRNA-seq

Raw sequencing data were processed using Cell Ranger (10x Genomics). Reads were aligned to the mouse reference genome (mm10), and gene expression count matrices were generated for each sample. Subsequent analyses were performed in R version 4.1.2 (R Foundation for Statistical Computing, Vienna, Austria, http://www.R-project.org/) utilizing the Seurat package while retaining sample identity metadata [20]. Cells were filtered using Seurat with a minimum detection threshold of 200 genes per cell (min.features = 200), and genes detected in at least three cells were retained (min.cells = 3). For downstream analyses, cells were further filtered based on the following criteria: 1,000 < nFeature_RNA < 4,000 and percent.mt < 10%. These thresholds were selected to exclude low-quality cells, potential empty droplets, and cells with elevated mitochondrial transcript proportions indicative of cellular stress or apoptosis. Unique molecular identifier counts were implicitly controlled through these gene-based filtering criteria, effectively removing cells with extremely low or excessively high transcript complexity. Doublets were not explicitly identified using a dedicated doublet-detection algorithm; however, cells with abnormally high gene counts were excluded by the upper nFeature RNA threshold, thereby mitigating potential doublet contamination.

To account for potential batch effects across samples, data integration was performed using Seurat’s anchor-based integration framework. Briefly, integration anchors were identified across datasets using FindIntegrationAnchors with 100 dimensions (dims = 1:100), followed by data integration using IntegrateData with the same dimensionality. The resulting integrated object was employed for all downstream analyses, effectively minimizing batch-driven variation while preserving biologically meaningful differences between samples.

Normalized and log-transformed expression values were generated using Seurat. Highly variable genes were identified, followed by principal component analysis (PCA). The top 50 principal components were used to construct a shared nearest-neighbor graph, and clustering was performed. Uniform Manifold Approximation and Projection (UMAP) was generated solely for visualization and was not utilized for clustering. Cell-type annotation was performed using canonical immune marker genes, with the full marker list provided in Supplementary Table S1. All samples were processed using an identical experimental and computational pipeline.

### Differentially expressed gene analysis

Differentially expressed gene (DEG) analysis was conducted using the Wilcoxon rank-sum test, with DEGs defined as those exhibiting a log fold change greater than 0.6 and an adjusted *P* value below 0.05. Gene Ontology (GO) enrichment was assessed using Enrichr and ClusterProfiler software.

For GO enrichment analyses, we utilized the gene-set collection GO_Biological_Process_2023.gmt. Single-sample gene set enrichment analysis was performed by computing gene-set activity scores via the Gene Set Variation Analysis (GSVA) R package (v2.0.7) using the single-sample gene set enrichment analysis (ssGSEA) method. Subsequently, Z-score normalization was applied to the resulting enrichment scores to facilitate comparisons across cells and samples. To mitigate the inflation of statistical power at the single-cell level due to pooling, we conducted per-mouse pseudo-bulk DEG analysis, defining DEGs as those with a log fold change greater than 1.0 and an adjusted *P* value below 0.1.

### Pseudotime analysis

Pseudotime analyses were executed using Monocle3 (v1.3.7). No additional feature or ordering-gene selection was applied for trajectory inference; instead, all genes retained after quality control (12,847 genes) were utilized for dimensionality reduction to avoid bias from arbitrary gene selection and to ensure reproducibility. Specifically, PCA was performed using the preprocess_cds function (num_dim = 50), and the resulting low-dimensional representation was employed for UMAP embedding and subsequent principal graph learning. Principal graph learning was performed using Monocle3’s learn_graph() function, applying the default parameters to the UMAP embedding generated from PCA. The learned principal graph was subsequently used for cell ordering and pseudotime assignment.

### Ethical statement

This study adhered strictly to the guidelines established by the National Institutes of Health and received approval from the Chiba University Ethics Committee (approval numbers: A4-088, A5-122, and A6-119).

### Statistical analysis

Data are presented as mean ± standard error of the mean or as absolute numbers and percentages, as appropriate. Group differences were analyzed using an unpaired *t*-test or Mann-Whitney *U* test, depending on the outcomes of the D’Agostino-Pearson omnibus normality test. Survival analysis was conducted using the log-rank test. All statistical analyses were performed using GraphPad Prism 10 (GraphPad Software, San Diego, CA, USA), with statistical significance defined as p<0.05. DEG analysis for scRNA-seq was performed utilizing Seurat with Benjamini–Hochberg false discovery rate (FDR) correction. GO and ssGSEA enrichments were reported using FDR-adjusted *P* values. Comparisons of cytokines were analyzed without multiple-testing correction due to the limited number of cytokines assessed.

## Results

### MSC treatment improves survival in a murine sepsis model

Following CLP-induced sepsis, mice were randomized to receive either MSCs or a vehicle control intravenously, with both groups receiving subcutaneous antibiotics according to our established protocol (Figure S1A). MSC treatment significantly improved survival rates compared to controls (*P* ═ 0.020), with a survival rate of 69.2% (9/13) in the MSC-treated group versus 33.3% (5/15) in the control group, 168 hours post-CLP surgery (Figure S1B). Two animals in the MSC group were excluded due to technical failure in MSC administration, which did not affect the interpretation of the survival analysis.

### Changes in systemic cytokine levels following sepsis induction

To investigate the alterations in cytokine profiles after MSC administration, blood cytokine levels were measured. At 6 hours post-CLP surgery, no significant differences were observed in plasma cytokine concentrations, including IL-1α, IL-1β, IL-6, IL-10, IL-12 p70, IL-13, TNF-α, IFN-γ, GM-CSF, MCP-1, MIP-1α, and MIP-1β, between the two groups (uncorrected *P* > 0.05 for all analyses) (Figure S2). These results suggest that systemic cytokine modulation may not be the primary mechanism underlying MSC-mediated protection in this model.

### scRNA-seq reveals cellular heterogeneity in peripheral blood after MSC treatment

**Figure 1.**
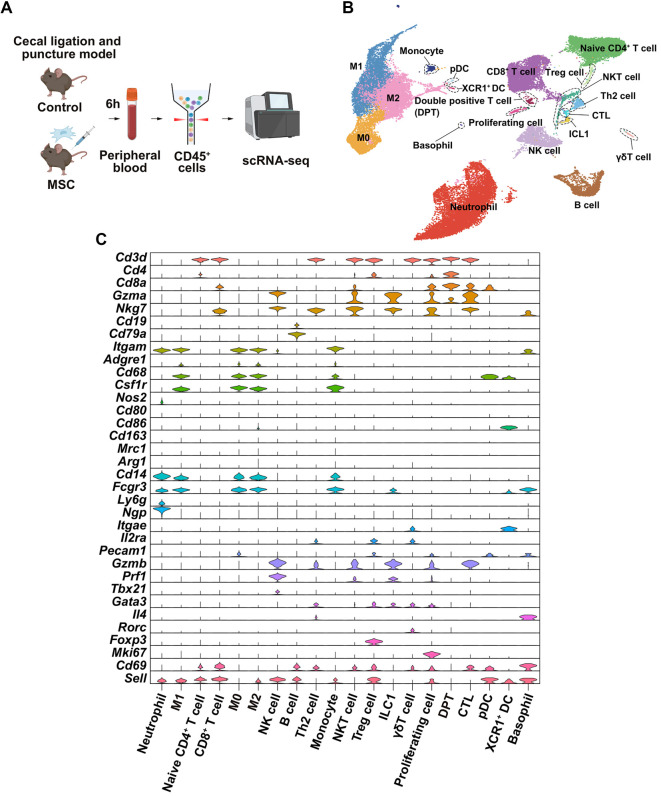
**scRNA-seq of peripheral-blood immune cells after MSC treatment in a murine sepsis model.** (A) Experimental overview and scRNA-seq workflow. Mice underwent CLP and received MSC or vehicle. Peripheral blood was collected 6 h later, CD45^+^ leukocytes were enriched, and scRNA-seq libraries were generated. (B) UMAP embedding of 41,052 integrated CD45^+^ cells from control and MSC-treated mice (*n* ═ 3 per group), resolving 20 immune populations. Clusters include neutrophils, basophils, monocytes, macrophage states (M0, M1, M2), B cells, NK cells, ILC1, DC subsets (pDC and XCR1^+^ DC), and multiple T-cell subsets (naïve CD4^+^, CD8^+^, CTL, Th2, Treg, NKT, γ δT, and DPT), as well as a proliferating cell cluster. (C) Violin plots showing normalized expression of representative lineage and state marker genes across the 20 annotated clusters, supporting cluster identity assignment and highlighting activation/proliferation programs in specific subsets. Abbreviations: scRNA-seq: Single-cell RNA sequencing; MSC: Mesenchymal stem cell; CLP: Cecal ligation and puncture; UMAP: Uniform manifold approximation and projection; M0: Unpolarized macrophage; M1: Classically activated macrophage; M2: Alternatively activated macrophage; NK: Natural killer; ILC1: Type 1 innate lymphoid cell; DC: Dendritic cell; pDC: Plasmacytoid dendritic cell; XCR1: X-C motif chemokine receptor 1; XCR1^+^ DC: XCR1-positive dendritic cell; CTL: Cytotoxic T lymphocyte; Th2: T helper 2 cell; Treg: Regulatory T cell; NKT: Natural killer T cell; γ δT: Gamma delta T cell; DPT: Double-positive T cell.

**Figure 2.**
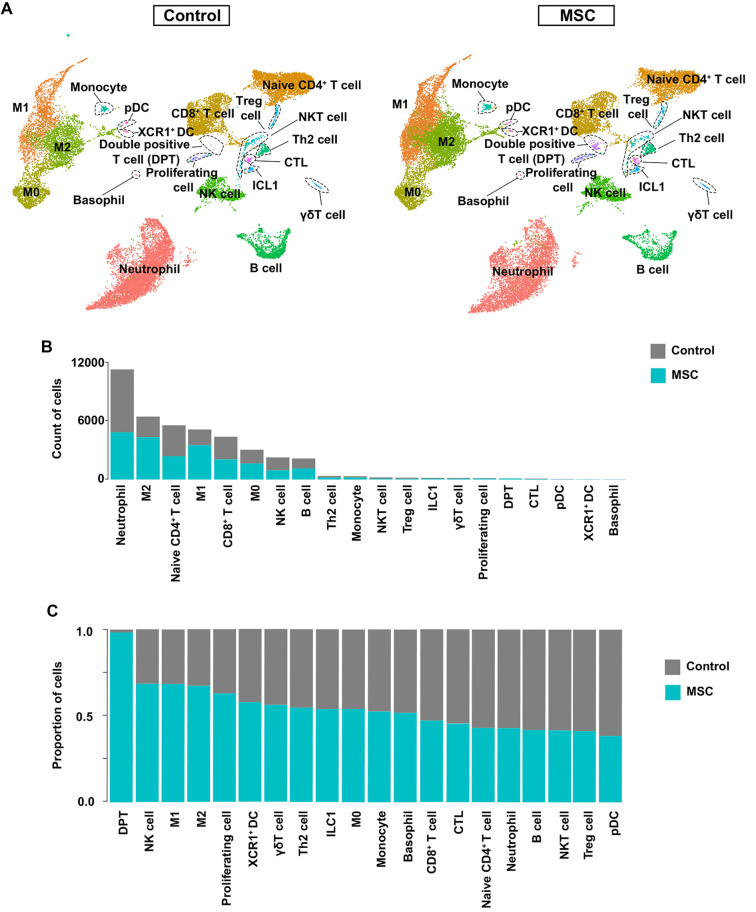
**MSC reshapes the peripheral-blood immune landscape after CLP.** (A) Separate UMAP embeddings of CD45^+^ peripheral-blood leukocytes profiled at 6 h after CLP, shown for control (left) and MSC-treated (right) mice. Cluster labels denote the immune populations identified in the integrated scRNA-seq dataset. (B) Bar plot showing the absolute number of cells assigned to each cluster in aggregated control (gray) and MSC (teal) samples, highlighting higher neutrophil, naïve CD4^+^ T-cell, and CD8^+^ T-cell counts in controls and increased M1/M2 macrophage representation with MSC treatment. (C) Stacked bar plot depicting the relative distribution of immune cell clusters within each group (control, gray; MSC, teal), showing enrichment of DPT, NK, M1/M2 macrophage, and proliferating-cell clusters following MSC treatment. Abbreviations: MSC: Mesenchymal stem cell; CLP: Cecal ligation and puncture; UMAP: Uniform manifold approximation and projection; scRNA-seq: Single-cell RNA sequencing; M0: Unpolarized macrophage; M1: Classically activated macrophage; M2: Alternatively activated macrophage; NK: Natural killer; ILC1: Type 1 innate lymphoid cell; DC: Dendritic cell; pDC: Plasmacytoid dendritic cell; XCR1: X-C motif chemokine receptor 1; XCR1^+^ DC: XCR1-positive dendritic cell; CTL: Cytotoxic T lymphocyte; Th2: T helper 2 cell; Treg: Regulatory T cell; NKT: Natural killer T cell; γ δT: Gamma delta T cell; DPT: Double-positive T cell.

To further elucidate the mechanisms of MSC action, we conducted scRNA-seq on CD45^+^ peripheral blood cells collected 6 hours after CLP and MSC or vehicle administration (Figure 1A). Using integrated datasets from three mice per group, we sequenced 41,052 cells. UMAP visualization revealed 20 distinct cell populations, including neutrophils, monocytes, and various macrophage subsets (M0, M1, and M2). Additionally, T-cell subsets such as CD4^+^, CD8^+^, regulatory T cells (Tregs), and T helper 2 (Th2) cells were identified, along with B cells, natural killer (NK) cells, and dendritic cells (Figure 1B and C and Figure S3).While neutrophils, naïve CD4^+^ T cells, and CD8^+^ T cells were more abundant in the control group, MSC treatment increased M1 and M2 macrophage populations (Figure 2A and B). The relative proportions of these cell populations differed between MSC-treated and control mice, with a higher proportion of double-positive T cells, NK cells, M1 and M2 macrophages, and proliferating cells in MSC-treated mice (Figure 2C). These findings suggest that MSC administration following sepsis induction selectively promotes the expansion of specific macrophage subsets in both number and proportion.

### MSC treatment induces transcriptional reprogramming in macrophage populations

**Figure 3.**
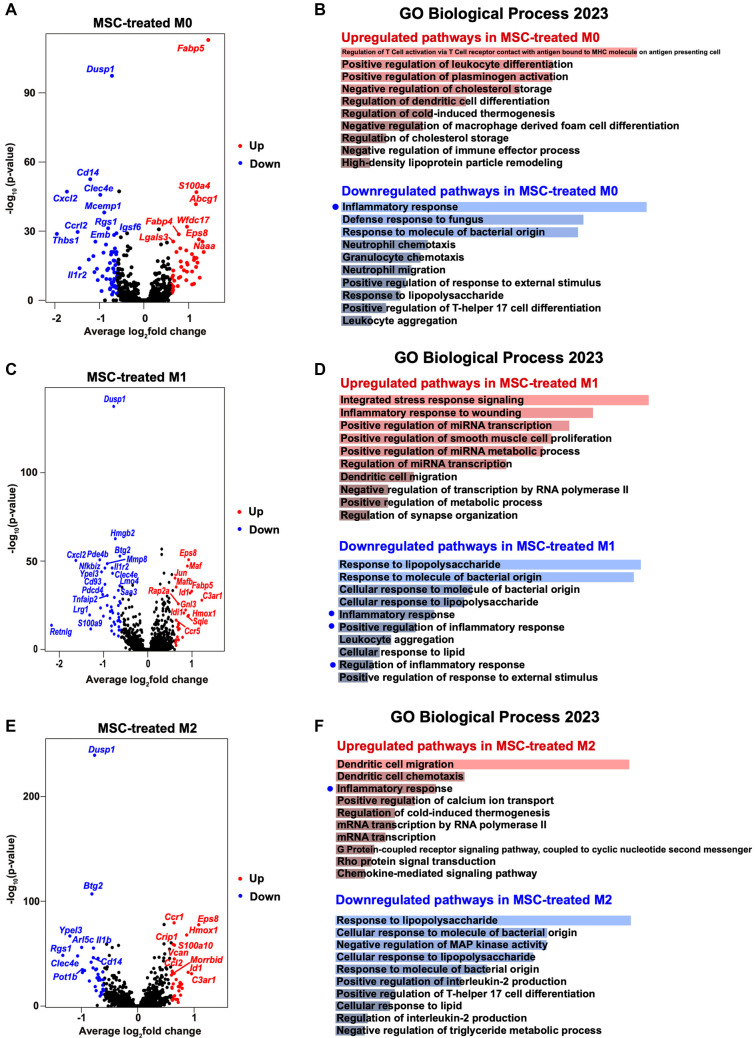
**MSC-driven transcriptional and pathway reprogramming across macrophage subsets.** (A, C, E) Volcano plots showing differential gene expression between MSC and control conditions within M0 (A), M1 (C), and M2 (E) macrophage clusters. The x-axis indicates average log2 fold change (MSC vs control) and the y-axis shows −log10(*P* value); upregulated genes are shown in red and downregulated genes in blue, with selected genes annotated. (B, D, F) GO Biological Process enrichment analyses for the upregulated (red) and downregulated (blue) gene sets in M0 (B), M1 (D), and M2 (F) macrophages. Bars denote the top enriched processes ranked by enrichment significance; blue dots highlight inflammation-related terms. Abbreviations: MSC: Mesenchymal stem cell; GO: Gene Ontology; scRNA-seq: Single-cell RNA sequencing; M0: Unpolarized macrophage; M1: Classically activated macrophage; M2: Alternatively activated macrophage.

Given the marked changes in the number and proportion of macrophage subsets, we focused on the transcriptomic alterations and polarization status of macrophages. GO enrichment analyses of DEGs in the M0, M1, and M2 macrophage clusters demonstrated that MSC treatment suppressed inflammatory pathways in M0 and M1 macrophages (Figure 3A–D and Figure S4). In M0 macrophages, MSC treatment resulted in the downregulation of inflammatory response genes and the upregulation of genes involved in T cell activation regulation, leukocyte differentiation, and the negative regulation of immune effector processes (Figure 3A and B). The most significantly downregulated genes included those involved in the inflammatory response to bacterial components (*Cd14*, *Cxcl2*, and *Clec4e*). Conversely, upregulated genes were primarily associated with cholesterol transport and metabolism (*Fabp5* and *Abcg1*) (Figure 3A).

Similarly, M1 macrophages from MSC-treated mice exhibited downregulation of pro-inflammatory genes and upregulation of stress-response genes (Figure 3C and D). Notably, downregulated genes included *Dusp1*, *Hmgb2*, *Cxcl2*, and *Pde4b*, while upregulated genes included transcription factors and regulators such as *Eps8*, *Maf*, *Jun*, and *Mafb* (Figure 3B). In contrast, M2 macrophages from MSC-treated mice showed upregulation of pathways associated with inflammatory regulation and immune activation, including *Hmox1* and *Ccr1*, and downregulation of pathways involved in bacterial response and IL-2 production (Figure 3E and F). Collectively, these findings suggest that MSC administration attenuates pro-inflammatory gene expression and enhances regulatory and metabolic pathways across macrophage subsets.

In the per-mouse pseudo-bulk DEG analysis, several signature genes common to M0, M1, and M2 were identified, consistent with the primary analysis. However, few pathways related to a downregulated inflammatory response in M1 or an upregulated inflammatory response in M2 were observed (Figure S5).

### MSCs promote M2-like polarization and suppress pro-inflammatory pathways

**Figure 4.**
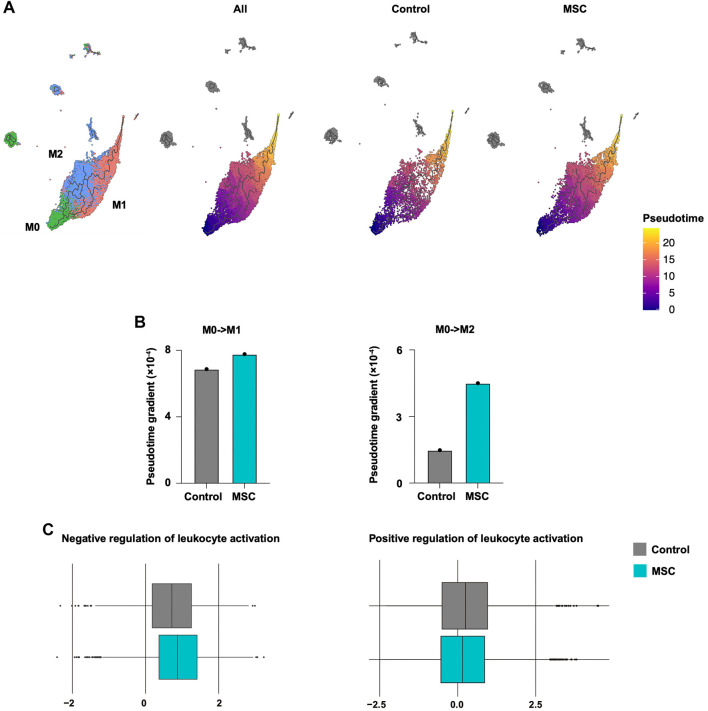
**MSC biases macrophage state transitions toward an M2-like program.** (A) Pseudotime trajectory inference for macrophage subsets (M0, M1, M2), illustrating a branching structure with progression from M0 toward M1 and M2 endpoints. Left, cluster annotation overlaid on the trajectory manifold; right, pseudotime coloring shown for all macrophages and stratified by group (control vs MSC), with increasing pseudotime indicated by the color scale. (B) Quantification of trajectory dynamics, shown as the pseudotime gradient along the M0→M1 and M0→M2 branches in control and MSC groups, highlighting an MSC-associated increase along the M0→M2 direction. (C) Box plots of ssGSEA scores for GO terms related to leukocyte activation within macrophage populations, showing increased enrichment of “negative regulation of leukocyte activation” and reduced enrichment of “positive regulation of leukocyte activation” in MSC compared with control. Abbreviations: MSC: Mesenchymal stem cell; ssGSEA: Single-sample gene set enrichment analysis; GO: Gene Ontology; M0: Unpolarized macrophage; M1: Classically activated macrophage; M2: Alternatively activated macrophage.

As inflammation induces macrophage activation and alters the distribution of their subsets, we performed trajectory analysis to investigate the functional changes associated with polarization [21, 22]. For pseudotime ordering, we defined the M0 cluster as the root state to anchor the trajectory and ensure interpretability of directionality. This analysis revealed that MSC treatment promoted a shift toward M2-like transcriptional states. The pseudotime gradient further demonstrated dynamic gene expression changes along the trajectory, with notable activation of M2-related gene sets (Figure 4A and B). ssGSEA further highlighted the upregulation of pathways associated with “negative regulation of leukocyte activation” and suppression of “positive regulation of leukocyte activation” (Figure 4C), while pathways related to “positive regulation of type 2 immune response” and “positive regulation of tissue remodeling” were not significantly affected (Figure S6).

### Transcriptional changes in inflammatory cytokines following MSC treatment in sepsis

To correlate plasma cytokine data with cellular transcription, we examined the expression of cytokine genes within the scRNA-seq dataset. In alignment with the multiplex assay, no significant increase in pro-inflammatory cytokine transcripts was detected at the population level in MSC-treated mice; however, subtle variations were evident in specific myeloid subsets (Figure S7). These results suggest that the effects of MSCs at 6 hours are more prominently reflected in pathway-level and polarization signatures than in overt cytokine overexpression.

## Discussion

**Figure 5.**
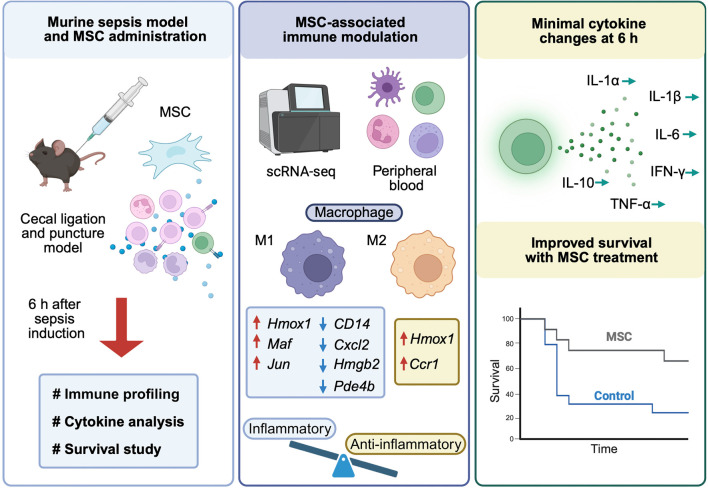
**Schematic summary of study design and MSC-associated immunomodulation in murine sepsis.** Overview of the CLP model with MSC administration and downstream readouts. Peripheral blood collected 6 h after sepsis induction was used for immune profiling by scRNA-seq and for plasma cytokine quantification, alongside a survival study. Integrated transcriptomic analyses highlight MSC-associated macrophage reprogramming toward an immunoregulatory, M2-like state, illustrated by increased expression of regulatory/stress-response genes (e.g., *Hmox1*) and reduced expression of inflammatory response genes (e.g., *Cd14*, *Cxcl2*, *Hmgb2*, *Pde4b*) within macrophage subsets. Despite these cellular transcriptional changes, systemic cytokine levels showed minimal early alterations at 6 h, whereas overall survival was improved in MSC-treated mice compared with controls. Arrows indicate the direction of MSC-associated gene expression changes. Abbreviations: CLP: Cecal ligation and puncture; MSC: Mesenchymal stem cell; scRNA-seq: Single-cell RNA sequencing; M1: Classically activated macrophage; M2: Alternatively activated macrophage; IFN-γ: Interferon gamma; TNF-α: Tumor necrosis factor alpha.

This study demonstrates that MSC treatment significantly enhances survival in a murine model of sepsis, accompanied by substantial transcriptional changes across diverse immune cell populations, particularly within macrophage subsets, despite minimal alterations in systemic cytokine levels. Notably, macrophages displayed enhanced immunoregulatory processes with M2-like polarization rather than an M1 phenotype, underscoring the immunomodulatory effects of MSCs (Figure 5).

Several recent single-cell studies have characterized immune responses in murine sepsis, including CLP models; however, they have not focused on MSC therapy or systemic CD45^+^ immune profiling [11–13]. In our study, scRNA-seq revealed that MSC administration following sepsis induction promoted an anti-inflammatory immune environment. Inflammatory markers such as *Cxcl2*, *Cd14*, and *Clec4e* were downregulated in M0 and M1 macrophages, which are typically associated with innate immune activation and excessive inflammation during sepsis [23–25]. This suppression may indicate a shift in macrophage polarization away from the pro-inflammatory M1 phenotype, potentially mediated by the immunomodulatory effects of MSCs. These findings corroborate prior studies that demonstrate MSCs exert anti-inflammatory effects, promote M2 functional polarization, mitigate the cytokine storm, and enhance survival in experimental sepsis models [9, 26–30]. Our study extends this knowledge by providing single-cell resolution of systemic immune responses, highlighting early transcriptional reprogramming of circulating macrophages and myeloid cells. The downregulation of these markers offers mechanistic insights into how MSC therapy modulates the innate immune landscape to facilitate the resolution of inflammation.

Pathway enrichment analysis further illustrated the functional reprogramming of macrophages associated with MSC administration. In M0 and M1 subsets, pathways related to inflammatory responses, lipopolysaccharide signaling, and chemotaxis were downregulated, while pathways associated with T-cell activation, leukocyte differentiation, and cholesterol biosynthesis were enriched. The latter may support the bioenergetic demands of activated immune cells during inflammation [31, 32]. Additionally, pathways linked to the integrated stress response were upregulated, potentially aiding cellular adaptation and inflammation resolution [33–35]. Conversely, in the M2 subsets, pathways related to the positive regulation of IL-2 production and negative regulation of mitogen-activated protein kinase activity were downregulated, while the inflammatory response pathway was upregulated. These findings indicate that macrophage polarization is not a linear transition toward a fixed functional state, but rather involves dynamic and subset-specific reprogramming of immune and inflammatory pathways.

Trajectory analysis suggested M2-like polarization rather than M1 phenotype following MSC treatment during sepsis. This observation was associated with the upregulation of *Hmox1*, a key anti-inflammatory and antioxidant gene, in both M1 and M2 macrophages. Previous studies indicate that MSCs facilitate this transition by enhancing mitophagy and reducing mitochondrial reactive oxygen species levels, thereby suppressing pro-inflammatory cytokine release and limiting pyroptotic cell death [36, 37]. The upregulation of *Hmox1* may enhance phagocytosis and bacterial clearance through its metabolites, including biliverdin, carbon monoxide, and bilirubin, which exert cytoprotective and immunomodulatory effects. Supporting this notion, the therapeutic benefits of MSCs have been shown to depend on *Hmox1*, as its inhibition significantly diminishes MSC efficacy in sepsis models [38]. Furthermore, M2 macrophages exhibited upregulation of pathways related to dendritic cell migration, chemotaxis, and RNA transcription. These findings suggest that MSCs not only reprogram macrophages toward a reparative phenotype but also enhance their interactions with other immune cells. This aligns with previous reports indicating that MSCs modulate immune responses through both paracrine signaling and direct cell–cell interactions [39–43]. Although these altered gene expressions are potentially linked to the pathophysiology of sepsis, the lack of validation limits definitive conclusions in this study. Future research is warranted to deepen these findings through validation and mechanistic approaches.

Despite significant transcriptional changes, systemic cytokine levels remained largely unchanged following MSC treatment. While we cannot exclude the possibility of undetected cytokine alterations at different time points or temporal fluctuations in cytokine dynamics [44, 45], we performed scRNA-seq to gain a deeper understanding of the transcriptomic effects of MSC administration post-sepsis induction. Our findings suggest that the protective effects of MSCs occur independently of systemic cytokine modulation and are likely mediated through targeted cellular reprogramming, as revealed by our transcriptomic analysis. Collectively, early MSC effects may be detectable at the level of cellular transcriptional programs before becoming evident in plasma cytokine profiles, an observation that warrants further investigation in time-course studies.

Previous studies have reported improved survival rates with MSC treatment in murine sepsis models, with efficacy varying by source (adipose tissue, bone marrow, umbilical cord blood), dosing regimen, and administration timing [7, 46–50]. Multiple MSC administrations have demonstrated greater cytokine modulation compared to single doses [51]. In our study, a single dose of adipose tissue-derived MSCs significantly improved survival in the CLP model. While administration timing (immediate vs. 1 hour post-surgery) has shown a minimal impact on efficacy, production conditions may influence the therapeutic potential of MSCs [52, 53]. Future research should focus on optimizing production protocols and exploring multiple dosing regimens to enhance MSC efficacy in sepsis treatment. MSC products have entered early-phase clinical trials for septic shock and acute respiratory distress syndrome, and the transcriptomic changes observed here provide a mechanistic rationale supporting their immunomodulatory potential. However, translating these findings to clinical practice will require clarification of optimal dose, timing, route of delivery, and an understanding of whether similar immune trajectories occur in patients. Integration with plasma biomarkers and immune phenotyping in clinical samples may bridge this gap.

This study presents several limitations. First, scRNA-seq and cytokine measurements were collected 6 hours post-CLP surgery, leaving the temporal dynamics of gene expression and cytokine profiles in later phases undetermined. Future research with extended cytokine profiling and time-course single-cell analysis is essential to fully characterize the temporal dynamics of MSC-mediated immunomodulation.

Second, validation of scRNA-seq findings through flow cytometry, immunohistochemistry, or qPCR was not possible due to the exhaustion of MSCs from the same production lot and financial constraints. Future studies should focus on validating the mechanisms underlying MSC-induced macrophage polarization.

Third, the pseudotime inference utilized reflects transcriptional similarity rather than direct lineage tracking; thus, it suggests but does not confirm a direct M1-to-M2 fate conversion. Therefore, our results should be interpreted as signatures of M2-like polarization temporally associated with MSC administration, rather than definitive evidence of mechanistic reprogramming.

Fourth, this study evaluated a single MSC product/source without direct comparisons to other MSC sources. Further investigations of other MSC types not included in this study could provide additional insights into the fundamental mechanisms underlying the therapeutic effects of MSCs.

Fifth, this exploratory study did not incorporate an a priori power calculation for survival outcomes, which may limit the robustness of effect size estimations. Additional limitations include the absence of a sham scRNA-seq baseline and the use of pooled sequencing, which restricts biological replication at the mouse level and could inflate statistical power at the cell level. Future work incorporating per-mouse sequencing or pseudo-bulk validation with larger sample sizes will be beneficial.

Sixth, this study was conducted exclusively in male mice, thus not assessing potential sex-specific differences in immune responses to MSC treatment, an aspect that should be addressed in future studies.

Seventh, while human-derived MSCs are known to exhibit low immunogenicity [15, 54], we did not directly evaluate their specific impact on host responses following administration. We did not track the biodistribution or persistence of MSCs post-infusion, nor did we assess potential immune recognition of the administered cells. Although human adipose-derived MSCs are generally considered hypo-immunogenic, the extent to which they engraft or interact with host immune cells during early sepsis remains unclear. Future studies employing MSC-tracking methodologies will be vital for elucidating these mechanisms.

Eighth, our analysis focused exclusively on circulating CD45^+^ cells, and peritoneal macrophages were not examined morphologically or phenotypically. Given that MSCs may influence local immune cell dynamics at the infection site prior to systemic effects, analyzing peritoneal macrophages represents a crucial next step for mechanistic investigation.

Ninth, the model was optimized to achieve approximately 50% mortality; however, our experiment recorded a control mortality of 66.7% at 168 hours. Variability in CLP severity is expected due to both technical and biological factors.

Finally, the role of MSC-derived extracellular vesicles, including exosomes, has not been addressed, despite reports indicating their potential to mediate anti-inflammatory effects in sepsis [55, 56]. Future research should aim to deepen our understanding of MSC-mediated immunomodulation and its influence on sepsis pathophysiology.

## Conclusion

This study demonstrated that MSC treatment significantly improved survival in a murine model of sepsis. scRNA-seq revealed that MSC treatment was associated with transcriptional changes indicative of M2-like macrophage polarization and a reduction in inflammatory signaling. These findings underscore the therapeutic potential of MSCs as immunomodulatory agents in the treatment of sepsis and highlight the value of single-cell transcriptomics in elucidating the complex cellular mechanisms underlying sepsis pathophysiology.

## Supplemental data

Supplemental data are available at the following link: https://www.bjbms.org/ojs/index.php/bjbms/article/view/13517/4139.

## Data Availability

The single-cell RNA-seq data generated for this study have been deposited in the Gene Expression Omnibus (GEO) under accession code GSE318688. Source data are provided with this paper.
